# Enhanced Photocatalytic Activity and Photoluminescence of ZnO Nano-Wires Coupled with Aluminum Nanostructures

**DOI:** 10.3390/nano12111941

**Published:** 2022-06-06

**Authors:** Mondher Rtimi, Nour Beydoun, Artur Movsesyan, Suzanna Akil, Sergei Kostcheev, Xavier Gassmann, Mohamed Lajnef, Radhouane Chtourou, Safi Jradi

**Affiliations:** 1 Laboratory Light, Nanomaterials & Nanotechnologies (L2n—CNRS-EMR 7004), University of Technology of Troyes, 10004 Troyes, France; mondher.rtimi@utt.fr (M.R.); nour.beydoun@utt.fr (N.B.); movsesyan@gmail.com (A.M.); sergei.kostcheev@utt.fr (S.K.); xavier.gassmann@utt.fr (X.G.); 2 Faculty of Sciences of Tunis, University of Tunis El Manar, Tunis 2092, Tunisia; 3 Research and Technology Centre of Energy, Borj Cedria B.P N° 95, Hammam-Lif 2050, Tunisia; mohamed.lajnef@gmail.com (M.L.); radhouane.chtourou@crten.rnrt.tn (R.C.); 4 LCP-A2MC Laboratory, Jean Barriol Institute, University of Lorraine, 1 Bd Arago, 57070 Metz, France; suzanna.akil@univ-lorraine.fr

**Keywords:** ZnO, aluminum, localized surface plasmons resonance (LSPR), photoluminescence, plasmonic photocatalysis, methylene blue

## Abstract

In this study, we fabricated a hybrid plasmonic/semiconductor material by combining the chemical bath deposition of zinc oxide nanowires (ZnONWs) with the physical vapor deposition of aluminum nanostructures (AlNSs) under controlled temperature and atmosphere. The morphological and the optical properties of the ZnONWs/AlNSs hybrid material fabricated at different temperatures (250, 350, and 450 °C) and thicknesses (5, 7, and 9 nm) of Al layers were investigated. By adjusting the deposition and annealing parameters, it was possible to tune the size distribution of the AlNSs. The resonant coupling between the plasmonic AlNSs and ZnONWs leads to an enhanced photoluminescence response. The photocatalytic activity was studied through photodegradation under UV-light irradiation of methylene blue (MB) adsorbed at the surface of ZnO. The MB photodegradation experiment reveals that the ZnONWs covered with 7 nm aluminum film and annealed at 450 °C exhibit the highest degradation efficiency. The comparison between ZnONws and ZnONws/AlNSs shows a photoluminescence enhancement factor of 1.7 and an increase in the kinetics constant of photodegradation with a factor of 4.

## 1. Introduction

ZnO-based materials can be found in several geometrical formations such as one-dimensional (nanorods, nanowires) [1] and two-dimensional structures (thin films, layers) [2], along with bulk crystals [3]. In this respect, ZnONWs have been widely studied because of their excellent mechanical flexibility, optical and electrical properties, and good environmental adaptability. Many techniques have been used to prepare ZnONWs, including physical techniques, such as vapor–liquid–solid (VLS) [4], chemical vapor deposition (CVD) [5], pulsed laser deposition (PLD) methods [6], and chemical techniques by hydrothermal [7] and chemical bath deposition (CBD) methods [8]. Nonetheless, the physical techniques usually require a lot of energy. In that context, the chemical techniques are reliable processes with low temperatures and low production cost that are facile and reproducible.

Due to the above-mentioned properties, ZnONWs were introduced as a promising material for photocatalysis applications. To enhance their photocatalytic activity and/or shift it to the visible range, several methods were introduced, such as dye sensitization [9], coupling with other semiconductors [10], and metal doping [11]. In this context, the coupling of ZnO with plasmonic noble metal nanoparticles (Au, Ag) was proposed to improve its photocatalytic performance [12,13,14]. In this case, the interaction between the light and metal nanoparticles creates waves at the surface of the metal called localized surface plasmon resonance (LSPR) [15,16]. Within this framework, Au-ZnO plasmonic hybrid structures were intensively exploited for light-induced pollutant degradation. The plasmonic response of the Au in the visible range induces an improvement in the charge carrier separation in the metal/semiconductor surface, which increases the number of electrons available at the ZnO conduction band and, in turn, increases the number of radical species, making the photodegradation reaction faster [17,18]. Furthermore, the size, shape, and morphology of Au nanoparticles (AuNPs) affect the LSPR as well as the photocatalytic performance [19]. The influence of AuNPs’ size on the photocatalytic activity of Au-ZnO hybrids was investigated by Kavitha et al. using ZnO nanorods (NRs) modified with AuNPs with diameters varying from 20 to 80 nm. Surprisingly, Au-ZnO nanohybrids with 40 nm AuNPs had better photocatalytic activity than those with smaller or bigger diameters. It is claimed that a “trade-off” mechanism exists between efficient charge transfer for tiny AuNPs and a higher LSPR impact for bigger AuNPs [20]. As well, to improve the photocatalytic activity in the visible range, coupling of ZnO with silver nanoparticles (AgNPs) is used. When AgNPs attach to a ZnO semiconductor, a Schottky barrier forms at the metal–semiconductor interface, and the absorption band of the hybrid structure shifts toward the visible range due to the combination between the Ag plasmonic effect and the ZnO defects. The fabrication of this metal–semiconductor hetero-junction is an effective method for improving charge carrier separation and enhancing photocatalytic efficiency in the visible range [14]. However, the high cost of the noble metals (Au, Ag) hinders their mass production and limits their exploitation. In this context, aluminum, which is the third most abundant element in the Earth’s crust behind oxygen and silicon, has great potential as a plasmonic material [21]. The use of aluminum to improve the optical properties of ZnO was investigated [22], and different applications were proposed, such as UV-photodetectors [23] and plasmonic lasers [24]. Several works introduced the Al doping of nanostructured ZnO to improve its photocatalytic activity [25,26]. It was found that Al−doped ZnO has better catalytic activities in dye degradation [27,28,29] and shows high electrochemical stability compared to pure ZnO [27]. The enhancement was attributed either to the increase in ZnO absorption [27], the contribution of Al^3+^ in the catalysis mechanism [28], or the increase in dye adsorption [29]. However, the reported studies do not investigate the plasmonic features, such as the size, shape, and thickness of Al nanoparticles and their effect on the photocatalytic performance of ZnO.

In this work, aluminum nanostructures (AlNSs) were introduced as plasmonic materiel to enhance the ZnONWs photocatalytic activity and photoluminescence (PL) emission. To obtain the plasmonic hybrid material, the ZnONWs were synthesized by a chemical bath deposition method and then decorated with AlNSs in a physical vapor deposition machine under controlled temperature and atmosphere. First, we deposited AlNSs on quartz to understand and control their plasmonic response. The same parameters were then applied to the deposition of AlNSs on ZnONWs. The influence of plasmonic features on the coupling between ZnONWs and AlNSs was then investigated by studying the PL response and photocatalytic activity of the hybrid system under UV light. The comparison between ZnONws and ZnONws/AlNSs shows a photoluminescence enhancement factor of 1.7 and an increase in the kinetics constant of photodegradation with a factor of 4. The PL and photocatalysis enhancements were discussed by considering the optical properties of AlNSs.

## 2. Materials and Methods

All chemicals and reagents, zinc acetate dehydrate ($\mathrm{Zn}{\left({\mathrm{CH}}_{3}\mathrm{COO}\right)}_{2}\;.\;2H_{2}O$), ethanolamine $\left(C_{2}H_{7}\mathrm{NO}\right)$, ethanol $\left(C_{2}H_{6}O\right)$, zinc nitrate hexahydrate ($\mathrm{Zn}{\left({\mathrm{NO}}_{3}\right)}_{2}\;.\;6H_{2}O$), hexamethylenetetramine ($C_{6}H_{12}N_{4}$), ammonia (${\mathrm{NH}}_{3}$), and methylene blue ($C_{16}H_{18}{\mathrm{CIN}}_{3}S$) were purchased from Sigma-Aldrich (Darmstadt, Germany) and used as received without further purification. ITO-glass substrates (30 ohms) were purchased from the company SOLEMS (Palaiseau, France).

The ZnONWs patterns were grown by CBD (chemical bath deposition) on ZnO seed layers. Aluminum nanostructures were deposited in a vacuum evaporator. The morphology and dimensions of the ZnONWs and ZnONWs/AlNSs patterns were characterized using a field-emission scanning electron microscope (SEM, Hitachi, SU-8030) (Hitachi High-Technologies Corporation, Tokyo, Japan) and an Atomic Force Microscope (AFM, BRUKER, Dimension ICON in “tscan asyst” mode or intermittent contact) (BRUKER Corporation, Billerica, MA, USA), and the images obtained were then analyzed by Nanoscope Analysis v.1.9 (soft) (BRUKER Corporation, Billerica, MA, USA). The optical characterizations were carried out with a UV-visible spectrometer (Varian Cary Scan 100, Seoul, South Korea) and spectrofluorometer (Horiba, Kyoto, Japan).

### 2.1. Preparation of the ZnO Seed Layers

The ZnO seed layers were prepared by sol–gel process using spin coating method on indium tin oxide–glass substrate (ITO-substrate). The ITO substrates were ultrasonically cleaned initially with ethanol and acetone in sequence for 10 min, then rinsed with ultrapure water, and finally dried in air [30]. The solution for the sol–gel reaction contained 0.75 M zinc acetate dehydrate ($\mathrm{Zn}{\left({\mathrm{CH}}_{3}\mathrm{COO}\right)}_{2};2H_{2}O$), and 0.75 M ethanolamine $\left(C_{2}H_{7}\mathrm{NO}\right)$ dissolved in absolute ethanol $\left(C_{2}H_{6}O\right)$. The obtained solution was stirred at 60 °C for 1 h to obtain a homogeneous and clear solution. After, the solution was aged for 24 h at room temperature. The ZnO seed layers were deposited on the ITO substrate using a spin-coater at 3000 rpm for 30 s. In this work, a single deposition iteration is used to obtain flat and homogeneous ZnO seed layers without ridges on the surface (Figure S1).

Finally, the obtained samples were annealed at 400 °C for one hour.

### 2.2. ZnO Nanowires Synthesis

The growth of the ZnONWs was completed using chemical bath deposition according to the protocol used by Syrrokostas et al. [31]. The precursor solution was prepared by dissolving 9.47 g of zinc nitrate hexahydrate ($\mathrm{Zn}{\left({\mathrm{NO}}_{3}\right)}_{2}\;.\;6H_{2}O$) and 7 g of hexamethylenetetramine HMTA ($C_{6}H_{12}N_{4}$) in 250 mL of ultrapure water; the final concentration was 0.2 M for each precursor. The obtained solution was stirred at 60 °C for one hour. The pH was adjusted to 9.5 by dropwise addition of ammonia (${\mathrm{NH}}_{3}$) as a chemical additive.

The ZnO seed layers were immersed in a bath containing the chemical precursor solution for 4 h. During that time, the solution was stirred at 90 °C. The ZnO nanowires formed by CBD were rinsed with deionized water and then annealed at 450 °C for 1 h in an oven.

### 2.3. Aluminum Nanostructures Fabrication

The fabrication of aluminum nanostructures was completed in 2 steps. First, aluminum layers of few nanometers were deposited on quartz substrates or on ZnONWs. The deposition was completed using Physical Vapor Deposition (PVD) machine “PLASSYS MEB400” (Plassys, Marolles-en-Hurepoix, France). The evaporation of aluminum is achieved by electronic bombardment under vacuum ($2\times 10^{-6}\;\mathrm{mBar}$). The film thickness was monitored by a quartz–crystal microbalance. The deposition rate was equal to $0.2\;\mathrm{nm}\cdot s^{-1}$. The aluminum deposition was performed on a beforehand heated substrate inside the evaporator, which is equipped with a heated sample holder controlled by a thermocouple temperature.

### 2.4. Photoluminescence Measurements

Photoluminescence measurements were carried out at room temperature and atmospheric air with LabRAM HR Evol Raman Spectrometer/HORIBA Scientific machine (Horiba, Kyoto, Japan).The excitation source was a continuous laser emitting at 325 nm/25 mW. The emission was detected using a CCD camera via a UV-objective (Thorlabs LMU-10x-NUV/NA 0.23) (Thorlabs, Newton, NJ, USA). The PL measurement was performed from 350 nm to 450 nm.

### 2.5. Methylene Blue Photocatalysis Experiments

The photocatalytic activity of ZnONWs and ZnONWs/AlNSs hybrid structures was evaluated by following the degradation reaction of methylene blue (MB) adsorbed at the ZnO surface. The adsorption of MB was completed by immersion of the sample in a beaker containing a volume of 1 mL of the MB solution ($C_{MB}=10^{-5}\;M$) for 15 min to establish the adsorption–desorption equilibrium. After that, the sample was placed at the bottom of a beaker containing 1.2 mL of the MB solution. The catalytic experiments were completed on ZnONWs deposed on a substrate and decorated with AlNSs. The quantity of ZnONWs was estimated to be about 2.22 mg. The quantity of aluminum deposited on the ZnONWs depended on the layer thickness. For 5 nm thickness (7 and 9 nm, respectively), the quantity is about $11.25\times 10^{-4}$ mg ($15.75\times 10^{-4}$ mg and $20.25\times 10^{-4}$ mg, respectively). Then, the photocatalytic activity was investigated under ultraviolet irradiation using Hamamtsu spotlight source (mercury–xenon lamp) with 4500 mW/cm^2^ intensity using a mercury line filter of 365 nm from Semrock (Hg01-365-25) (Hamamtsu, Japan). The distance between the UV lamp and the sample was about 5 cm. To follow the MB degradation, the absorption of the solution was measured at 610 nm every 5 min using a CARY UV-visible spectrometer (Varian Cary Scan 100, Seoul, South Korea).

## 3. Results and Discussion

### 3.1. Parametric Study of the Aluminum Nanostructures Fabrication

**Figure 1:**
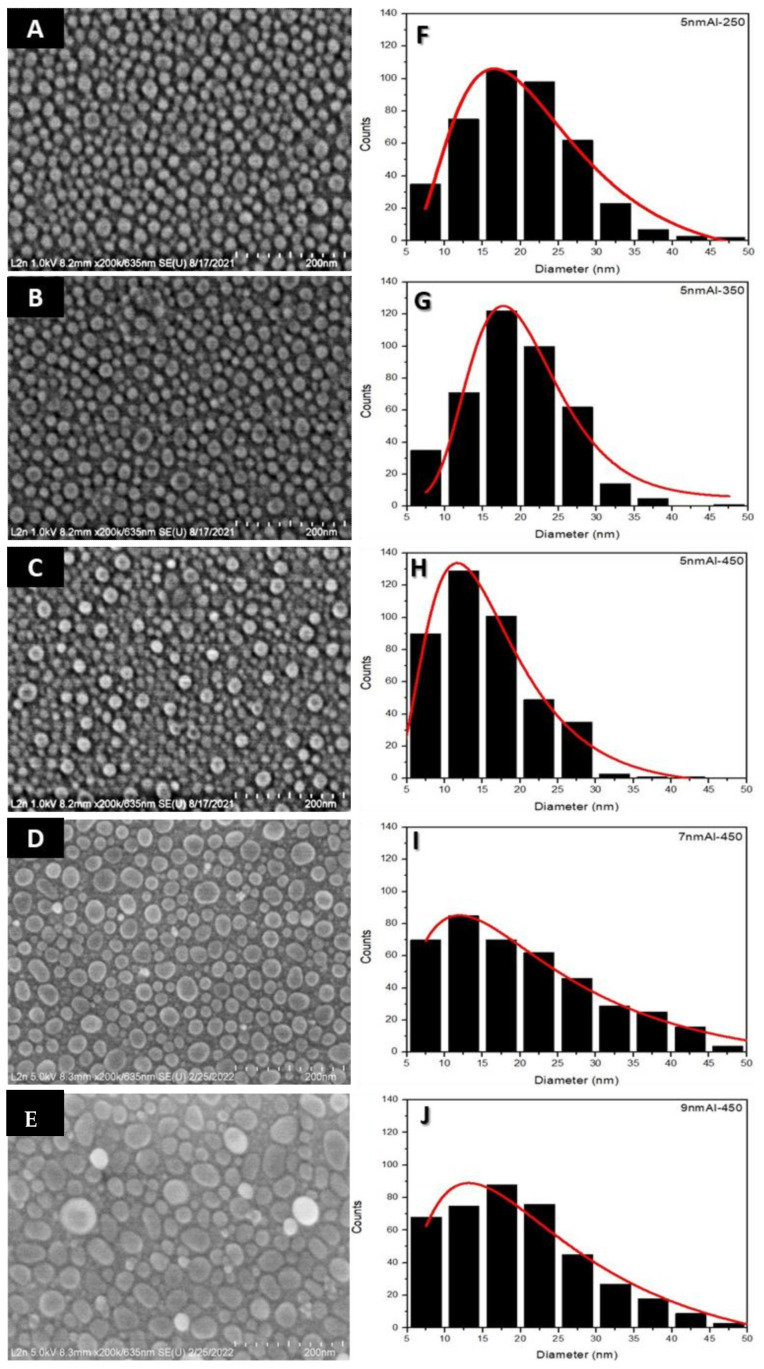
SEM images of Al films deposited by PVD on quartz substrate under different experimental conditions and their diameter distributions. (**A**–**C**) SEM images of 5 nm of aluminum film deposited at 250, 350, and 450 °C, respectively. (**D**,**E**) SEM images of 7 and 9 nm of aluminum film, respectively, deposited at 450 °C. The figures (**F**–**J**) are the histograms of the diameter distribution obtained from the SEM images (**A**–**E**), respectively. The histograms were fitted by a logNormal function to find the mean diameter $d_{m}$.

**Figure 2:**
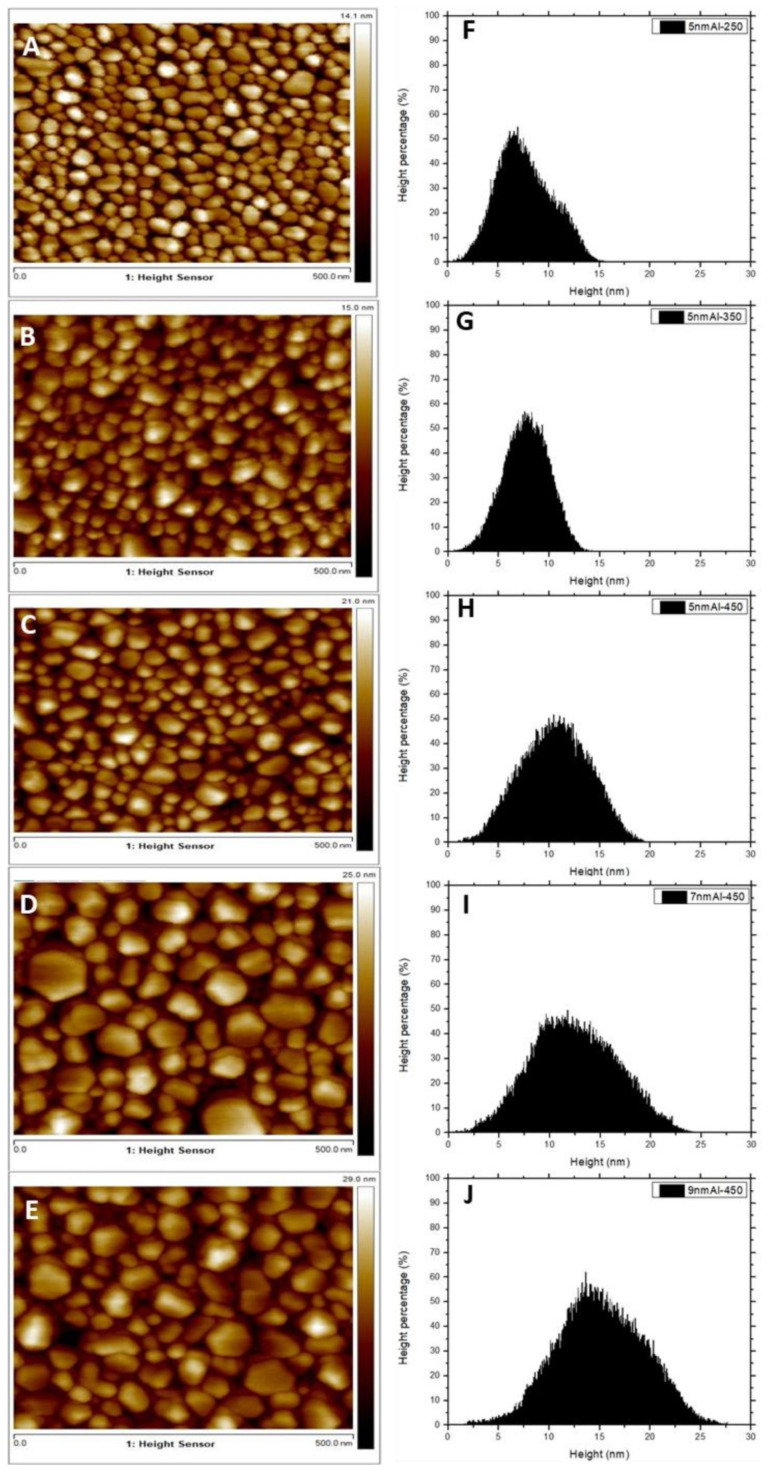
(**A**–**E**): AFM images of 5 nm Al−250, 5 nm Al−350, 5 nm Al−450, 7 nm Al−450, and 9 nm Al−450, respectively, fabricated on quartz substrate, and (**F**–**J**) their corresponding height histograms.

Figure 1 presents the SEM images of AlNSs deposited on quartz substrates under different experimental conditions and their mean diameter histograms. Figure 1A–C shows SEM images of 5 nm of aluminum deposited at 250, 350, and 450 °C, respectively. Figure 1D,E shows AlNSs deposited at 450 °C with a thickness of 7 and 9 nm, respectively. To determine the average diameter of the AlNSs, the SEM images were processed by ImageJ software; the histogram results are shown in Figure 1F–J for each experimental condition. The histograms were fitted by a logNormal function to find the mean diameter $d_{m}$. The AFM images of the same samples and their corresponding depth histograms are presented in Figure 2. The average sizes of the AlNSs and their average height for different experimental deposition conditions are given in Table 1.

The average diameter for the 5 nm Al samples is less than 50 nm; it decreases with temperature. It is about 17, 17.5, and 11.5 nm for the 5 nm Al−250, 5 nm Al−350 and 5 nm Al−450 samples, respectively (Figure 1A–C,F–H). By comparing the samples deposited at 250 °C and 450 °C, it can be seen that the number of AlNSs smaller than 15 nm increases, and of AlNSs larger than 30 nm drastically decreases. When the Al thickness deposited at 450 °C increases to 7 and 9 nm (Figure 1D,E,I,J), the average size of AlNSs increases, and $d_{m}$ is about 12 and 13.5 nm, respectively. The AFM images (Figure 2) show that the shape of AlNSs is almost cylindrical and not uniform. Furthermore, the average height of the NSs increases with temperature and deposition thickness. The corresponding height is 6.5, 7.5, 10.5, 13, and 14.5 nm for the 5 nm Al−250, 5 nm Al−350, 5 nm Al−450, 7 nm Al−450, and 9 nm Al−450 samples, respectively (Figure 2F–J). The corresponding shape factor tends to be one, as calculated in Table 1. This phenomenon can be explained by the migration and cumulation of the matter under the effect of annealing, which leads to the formation of the AlNSs [32]. For the 7 nm and 9 nm Al−450 samples, it can be clearly seen that the size and height of AlNSs grow, and the shape factor is around one.

**Figure 3:**
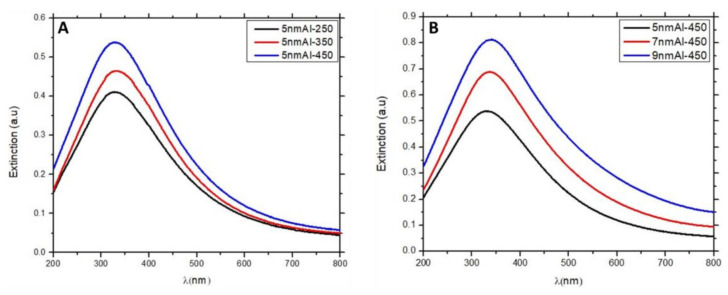
UV-visible extinction spectra of AlNSs. (**A**) 5 nm Al films annealed at 250, 350, and 450 °C. (**B**) 5, 7, and 9 nm Al films annealed at 450 °C.

Figure 3 shows the UV-visible extinction spectra of the AlNSs deposited on a quartz substrate. The extinction band is attributed to the localized surface plasmon resonances (LSPR) [32] of aluminum. This phenomenon is assigned to the collective and coherent oscillations of the surface conduction electrons of the aluminum nanostructures [33]. Figure 3A presents the extinction spectra of 5 nm Al films deposited at 250, 350, and 450 °C. The extinction peak of the 5 nm Al−250, 5 nm Al−350, and 5 nm Al−450 is located at 333, 330, and 326 nm, respectively (Figure 3A). The intensity of the LSPR band grows with temperature, and a slight blue shift of 7 nm is observed between 5 nm Al−250 and 5 nm Al−450. To understand this slightly small shift of peak positions, we need to consider several factors. Indeed, for the 5 nm Al−450 extinction, we are expecting a larger blue shift than 7 nm since the average diameter decreased and the height simultaneously increased. With the height increase, the number of charges is augmented, and with the diameter decrease, the separation between them is decreased. As a result, the displacement of the electron gas experiences a stronger restoring force, which leads to shorter wavelength resonances. Nonetheless, in our system, we need to look at the problem more globally. In coupled systems, the plasmon resonances strongly depend on the interparticle distance. The resonant wavelength of bright modes is redshifted with the decrease of interparticle distance. The strong redshift in closely packed nanoparticles is dictated by the large energy splitting of low-energy bright and high-energy dark modes. Since the AlNSs in 5 nm Al−450 have a shorter interparticle distance than 5 nm Al−250, the resulting resonant wavelength of the bright mode will be largely redshifted compared 5 nm Al−250. To summarize, for the 5 nm Al−450 sample, the plasmon resonance should be blueshifted compared 5 nm Al−250 due to a bigger height and smaller diameter; however, this blueshift is compensated by a shorter interparticle distance.

The interplay between the height and the interparticle distance leads to close plasmon peak positions for three samples (5 nm Al−250, 5 nm Al−350, and 5 nm Al−450). The increase in the extinction amplitude of the thick AlNSs can be explained by the fact that with the increase in the height, volume/surface ratio, the matter that can be oxidized with the interaction with the air is reduced.

Figure 3B shows the extinction spectra of different thicknesses of AlNS samples deposited at 450 °C (5 nm Al−450, 7 nm Al−450, and 9 nm Al−450, respectively). The intensity of the LSPR band increases with the thickness of Al film, and a slight red shift of 8 nm is observed. Here, again, if we consider the average size increase from 12 to 16 nm (5 nm Al−450 and 9 nm Al−450, respectively), one can expect a larger redshift. However, this expected redshift is compensated with the height increase from 10.5 to 14.5 nm for the same samples and the fact that the aluminum nanostructures prepared at different temperatures are not uniform (Figure 2).

### 3.2. Morphology of ZnONws and ZnONws Covered by the Aluminum Nanostructures

**Figure 4:**
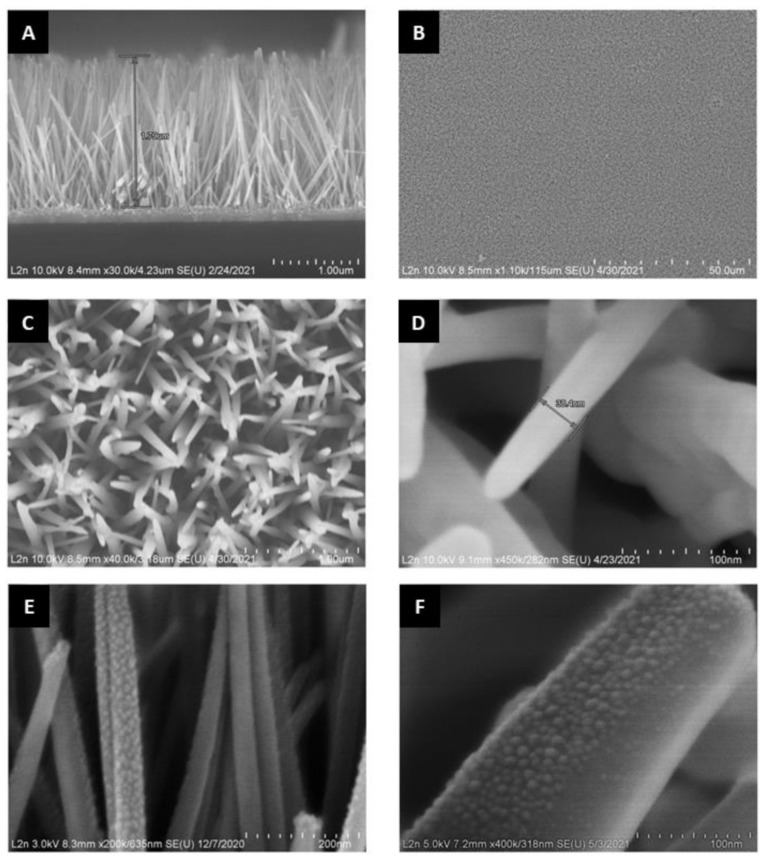
SEM images of ZnONws sample and ZnONws/AlNSs hybrid structure. (**A**) Cross-section of ZnONws sample. (**B**–**D**) Seen from the front with different magnifications of ZnONws sample, (**E**) ZnONws/AlNSs (5 nm; 450 °C) and (**F**) zoom on a nanowire of ZnO covered with AlNSs.

Figure 4 shows SEM images of ZnONWs sample grown by the chemical bath deposition method on the seed layers deposited on the ITO-glass substrate and ZnONWs/AlNSs (5 nm; 450 °C) hybrid structure. It can be seen from the cross-section realized in Figure 4A that the ZnONWs sample consists of straight-line morphology almost perpendicular to the surface of the substrate, and the length of these nanowires is about 1.8 μm.

Figure 4B shows an overall view of the sample and confirms the homogeneity of the nanowire distribution on the substrate. Figure 4C,D confirms that nanowires have a cylindric shape, smooth, flat, and sleek surface, and the diameter of the nanowire is about 50 nm.

Figure 4E,F shows the surface of the ZnO nanowire after deposition of 5 nm Al film and annealing at 450 °C. The surface of the ZnONWs/AlNSs becomes rough, contrary to the pure ZnONWs, which confirms the attachment of the aluminum nanostructures to the zinc oxide nanowires to form the plasmonic hybrid structure. The average size of the AlNSs on ZnONWs is nearly the same as on the quartz substrate (~12.5 nm). On the other hand, the upper part of the nanowires is completely covered by the AlNSs, while the lower part is partially covered (Figure 4F).

### 3.3. Optical Properties

#### 3.3.1. UV-Visible Response

**Figure 5:**
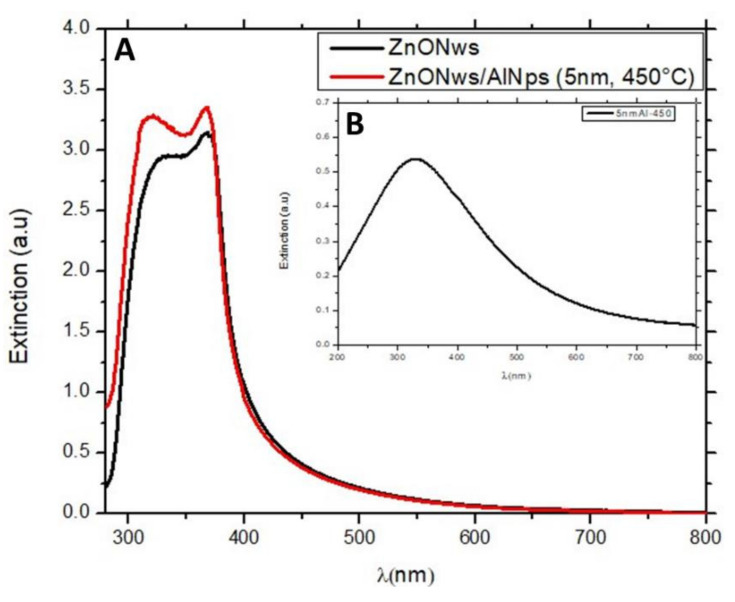
UV-visible extinction spectra of (**A**) ZnONws and ZnONws/AlNSs (5 nm; 450 °C) and (**B**) 5 nm Al–450 sample.

UV-visible extinction spectra measurements of ZnONWs and ZnONWs/AlNSs (5 nm; 450 °C) were carried out in the wavelength range of 280 nm–800 nm, as presented in Figure 5. These extinction spectra show a peak at around 367 nm that is related to the direct transitions of electrons from the valence band to the conduction band in ZnONWs. The ultraviolet absorption for the ZnONWs/AlNSs hybrid structure increases because of the AlNSs LSPR band.

Figure S2 revealed that the $E_{g}$ (band gap energy) of the ZnONws is 3.20 eV, and that of the ZnONws/AlNSs hybrid structure is 3.22 eV. This energy shift can be explained by the fact that LSPRs affect the free excitons concentration in the conduction band of the ZnO due to exciton–plasmon interactions (Burstein–Moss shift) [34,35].

#### 3.3.2. Photoluminescence Response

Photoluminescence (PL) response is an excellent method to study the edge band gap transition and the excitonic structures [36,37]. According to the literature, the near band edge emission response of ZnONWs is usually dominated by the emission from excitons localized by impurities, the donor–accepter pair, longitudinal optic, and the phonon replicas of the main transitions [38,39].

**Figure 6:**
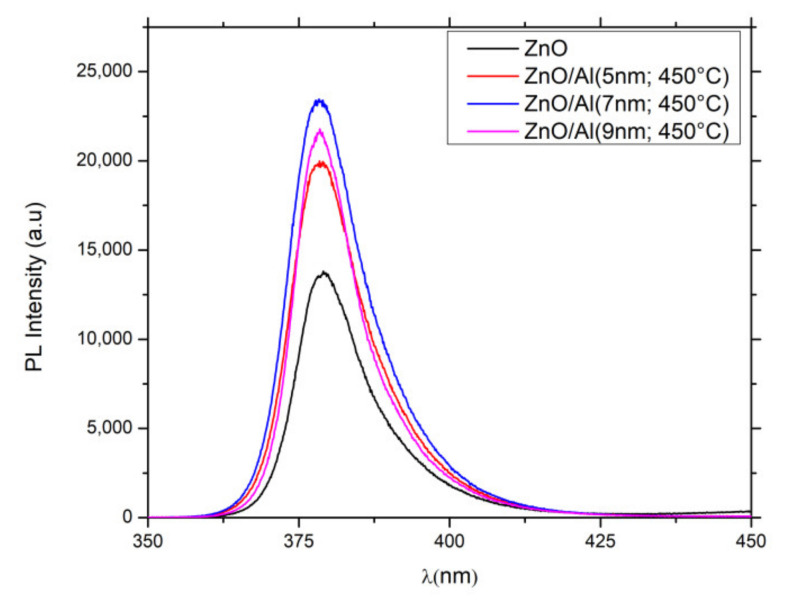
Photoluminescence spectra. ZnONws and ZnONws/AlNSs (5 nm, 7 nm, 9 nm; 450 °C).

To study the effect of the localized surface plasmon excited in AlNSs on the ZnONWs properties, PL measurements of bare ZnONWs and the ZnONWs/AlNSs hybrid structure with different thicknesses of Al films annealed at 450 °C were carried out. Figure 6 shows that the PL peak of ZnONWs and ZnONWs/AlNSs is located at 379.2 nm and 378.5 nm, respectively (Table 2). This slight blue shift in the presence of the AlNSs can be explained by the Burstein–Moss effect. We can notice that whatever the thickness of Al, the ZnONWs/AlNSs hybrid material leads to an enhancement of the PL signal compared to ZnONWs. The PL enhancement could be explained by the presence of Al nanoantennas coupled to ZnONWs via a well-known two mechanisms. The first is attributed to the strong electromagnetic field confined in the vicinity of AlNSs due to the excitation of plasmon resonances. This confined field increases the excitation rate of the ZnO located at that near-field of AlNSs. Secondly, AlNS may act as an optical resonator by increasing the relaxation rates (Purcell effect) in the emitter at its emission wavelength. This leads to a strong modification of the local density of states and of the ZnO emission rate due to the plasmon–exciton coupling.

The enhancement factor (EF) depends on the thickness of Al film (from 5 to 9 nm) deposited by PVD and annealed at 450 °C. EF increases from 1.45 for 5 nm Al to 1.70 for 7 nm and then drops down to 1.57 for 9 nm. This behavior may be explained by the incomplete dewetting of the Al layer when the thickness increases. The residual layer of Al would be responsible for the absorption of some part of the light, leading to a loss of energy during the excitation. The absorption increase in the overall spectral range can be indeed observed in Figure 3B, where the absorption at 325 nm increases from 0.53 for 5 nm Al−450 to 0.80 for 9 nm Al−450.

### 3.4. Photocatalytic Activity in the Presence of Methylene Blue

**Figure 7:**
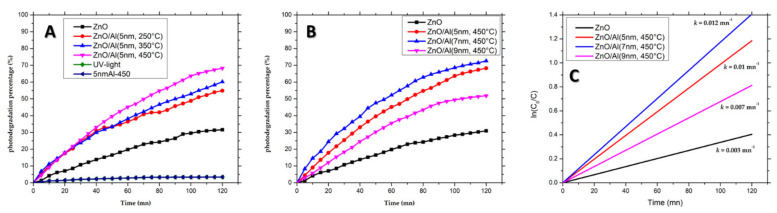
Photocatalytic activity of aluminum nanostructures deposited on ZnONws samples under UV-light irradiation (λ = 365 nm). (**A**) ZnONws, ZnONws/AlNSs (5 nm; 250, 350, and 450 °C), MB under UV-light, and 5 nm Al−450 sample. (**B**) ZnONws and ZnONws/AlNSs (5, 7, and 9 nm; 450 °C). (**C**) The plot of $ln\left(C_{0}/C\right)$ versus time to calculate the pseudo-first-order of kinetic degradation of ZnONws and ZnONws/AlNSs (450 °C; 5, 7, 9 nm) samples.

The experimental results of the photodegradation of MB by ZnONWs and hybrid structures are shown in Figure 7A–C presents the plot of the pseudo-first-order of the kinetic degradation rate. Figure 7A shows the photodegradation rate of the MB dye in the presence of ZnONWs, ZnONWs/AlNSs (5 nm Al film annealed at 250, 350, and 450 °C), 5 nm Al−450 sample and under UV-light. One can note that whatever the annealing temperature, the photodegradation rate is higher for ZnONWs/AlNSs as compared to bare ZnONWs. ZnONWs/AlNSs (5 nm; 450 °C) show the highest degradation rate (69%).

However, the effect of annealing temperature seems to be slight and less significant than that of Al thickness. Figure 7B shows the photodegradation rate profile as a function of time for the same samples presented in Figure 6. The ZnONWs/AlNSs (7 nm; 450 °C) hybrid structure presents the highest photodegradation rate of 74% after 120 min of irradiation, while the sample with ZnONWs alone led to only 30% degradation for the same irradiation time. For the hybrid-structure ZnONWs/AlNSs (5 nm and 9 nm; 450 °C), the photodegradation rates were 70% and 52% after 120 min irradiation time, respectively. For further exploration, the Langmuir–Hinshelwood (L–H) model was used to determine the methylene blue dye’s degradation rate constant [40,41,42,43]:

(1) $$ln\left(\frac{C_{0}}{C}\right)=k\times t$$

where $C_{0}$: concentration initial of MB; $C_{\;}$: concentration of MB after illumination time *t*; k: pseudo-first-order of kinetic rate degradation (rate constant).

All curves (Figure 7C) show an almost linear aspect, which indicates that MB dye degradation follows pseudo-first-order reaction kinetics. From the plot of $ln\left(\frac{C_{0}}{C}\right)$ versus time, the rate constant *k* was determined from the slope of the straight line. The calculated rate constants *k* are 0.003, 0.01, 0.012, and 0.007 $\min^{-1}$ for ZnONWs, ZnONWs/AlNSs (5 nm; 450 °C), ZnONWs/AlNSs (7 nm; 450 °C), and ZnONWs/AlNSs (9 nm; 450 °C), respectively. A higher first-order constant indicates more photocatalytic activity. Therefore, ZnONWs/AlNSs (7 nm; 450 °C) hybrid structure presents the highest photocatalytic activity, and the corresponding k is four times higher than that of the ZnONWs structure. One can note that k drops down when the thickness increases from 7 to 9 nm. This behavior, already observed with the PL measurements, could be explained by the incomplete dewetting of the relatively thick Al layer. This continuous layer of Al would be responsible for the absorption of light, reducing the number of photons absorbed by ZnO.

The significant improvement in the photocatalytic activity for the ZnONws with aluminum nanostructures can be explained by the plasmonic coupling between the AlNSs and the ZnONWs. Indeed, the extinction spectra of AlNSs and the excitonic band of ZnO are overlapped (Figure 5). So, during the excitation with the spotlight source at 365 nm, both the LSPR of AlNSs and the excitonic peak of ZnO are excited. The absorbed light at the plasmon wavelength generates a non-equilibrium distribution of electrons, which can decay in the generation of hot electrons on the surface of the nanostructure or low-energy electrons inside the nanostructure [40]. The generation of the latter ones results in heat in metals. Hot electrons’ generation and their injection into the conduction band of the semiconductor [41] and photothermal heating [42] can significantly increase photocatalytic activity. The photoluminescence enhancement, as shown in Figure 6, confirms that there is a direct resonant coupling between the zinc oxide excitons and the localized surface plasmons in the AlNSs.

**Figure 8:**
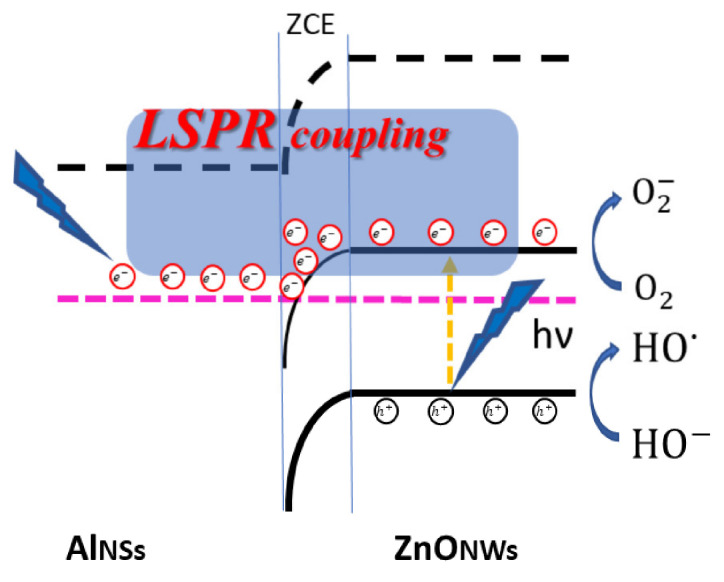
Scheme to illustrate the coupling between ZnONWs and AlNSs in photocatalysis.

Regarding the photocatalysis process, the MB dye is adsorbed on the ZnONW/AlNS hybrid structure (Equation (2)); then, under the UV illumination and the plasmonic coupling between the ZnONWs and the AlNSs, an electron ($e^{-}$) in the valence band of the ZnONWs is excited to the conduction band, leaving behind a positive hole ($h^{+}$) (Equation (3)). These electrons and holes will make several reactions (Figure 8) (Equations (4)–(7)) to produce HO*, which is responsible for the degradation of the methylene blue dye (Equation (8)). Thus, plasmonic coupling in the ZnONws/AlNSs hybrid structures increases the number of electrons available at the ZnO conduction band, which, in turn, increases the number of radical species (${\mathrm{HO}}^{\cdot }$), making the photodegradation reaction faster and the solution rapidly become colorless.

MB (in the solution) + ZnONws/AlNSs → MB-ZnONws/AlNSs (Adsorption)(2)

(3) $$\mathrm{MB}-\mathrm{ZnONws}/\mathrm{AlNSs}+h\nu \;\to \;e^{-}+h^{+}$$

(4) $$e^{-}+O_{2}\;\to \;O_{2}^{-}$$

(5) $$h^{+}+{\mathrm{HO}}^{-}\;\to \;{\mathrm{HO}}^{\cdot }$$

(6) $$e^{-}+O_{2}^{-}+2H^{+}\;\to \;H_{2}O_{2}$$

(7) $$e^{-}+H_{2}O_{2}\;\to \;{\mathrm{HO}}^{-}+{\mathrm{HO}}^{\cdot }$$

(8) $$\mathrm{MB}\;\mathrm{Dye}+{\mathrm{HO}}^{\cdot }\;\to \;{\mathrm{CO}}_{2}+H_{2}O+\mathrm{Other}\;\mathrm{photodegradation}\;\mathrm{products}\;$$

**Figure 9:**
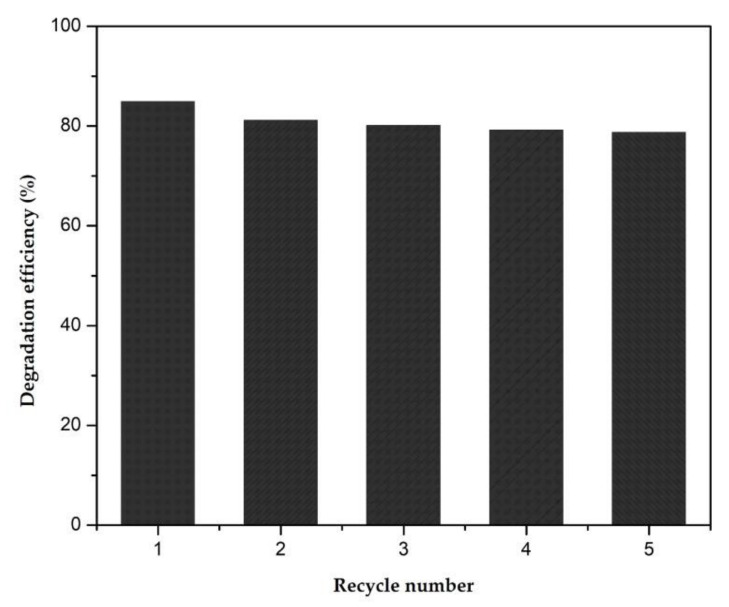
Recycling test of ZnONWs/AlNSs (7 nm; 450 °C) photocatalyst for MB dye.

To evaluate the stability of the produced ZnONWs/AlNSs (7 nm; 450 °C) hybrid structure photocatalyst, recycling experiments were carried out under similar conditions. Photodegradation experiments were repeated for five cycles with the same sample. The sample was thoroughly washed after each experiment. The results from the recycling runs are shown in Figure 9. The degradation efficiency decreased slightly after five cycles. The catalyst still exhibited more than 78% activity towards MB. Therefore, the hybrid nanocomposites are efficient and stable photocatalysts with the potential for reuse in degradation 

## 4. Conclusions

In this work, zinc oxide nanowires were synthesized by a chemical bath deposition method and then decorated with aluminum nanostructures deposited in a physical vapor machine to obtain the ZnONws/AlNSs hybrid structure. The plasmonic response of the AlNSs depends on the deposition temperature and aluminum thickness. The resonant coupling between the ZnONW excitons and the localized surface plasmons in the AlNSs increased the photoluminescence by 1.7 times compared to ZnONWs alone. It was also found that this hybrid plasmonic structure enhanced the kinetics of methylene blue degradation reaction fourfold as compared to the zinc oxide alone.

## Figures and Tables

**Table 1:** The average diameter ($d_{m}$), height (h), and shape factor of AlNSs calculated, respectively, from the SEM, AFM images, and the ratio $d_{m}/h$.

	Average Size $d_{m}$ (nm)	Average Height h (nm)	Shape Factor $d_{m}/h$
5 nmAl−250	17	6.5	2.6
5 nmAl−350	17.5	7.5	2.3
5 nmAl−450	11.5	10.5	1.1
7 nmAl−450	12	13	0.9
9 nmAl−450	13.5	14.5	0.9

**Table 2:** Details of PL-peak parameters and PL-intensity ratio of each sample compared to ZnO sample.

	Centre (nm)	$I_{0}$ (a.u)	PL Enhancement Factor
ZnONws	379.2	13,704	1
ZnONws/AlNSs (5 nm)	378.5	19,821	1.45
ZnONws/AlNSs (7 nm)	378.5	23,190	1.70
ZnONws/AlNSs (9 nm)	378.5	21,500	1.57

## Data Availability

The data are available upon request.

## Supplementary Materials

The following supporting information can be downloaded at: https://www.mdpi.com/article/10.3390/nano12111941/s1, Figure S1: SEM images for the ZnO seed layers with different cycle of deposition procedure. (A) ZnO seed layers deposited in 5 cycles; (B) ZnO seed layers deposited in 3 cycles; (C) ZnO seed layer deposited in 1 cycle; (D) Zoom on a ridge; (E) ZnONWs grown on seed deposited in 5 cycles; (F) Zoom on a seed layer deposited in 1 cycle.; Figure S2: Tauc plot of ZnONws and ZnONws/AlNSs.; References [44,45,46,47].
