# Supplementary material for: Enhanced Photocatalytic Activity and Photoluminescence of ZnO Nano-Wires Coupled with Aluminum Nanostructures
Source: Nanomaterials (Basel). 2022 Jun 6;12(11):1941. doi: 10.3390/nano12111941 (PMC9182769; doi:10.3390/nano12111941)
Supplement: Supplementary file 1 [file nanomaterials-12-01941-s001.zip › nanomaterials-1710195-supplementary.pdf]

nanomaterials

Supplementary Materials

Enhanced Photocatalytic Activity and Photoluminescence of ZnO Nano-Wires Coupled with Aluminum Nanostructures

Mondher Rtimi [1,2,3], Nour Beydoun [1], Artur Movsesyan [1], Suzanna Akil [4], Sergei Kostcheev [1], Xavier Gassmann [1], Mohamed Lajnef [3], Radhouane Chtourou [3] and Safi Jradi [1,*]

1 Laboratory Light, Nanomaterials & Nanotechnologies (L2n—CNRS-EMR 7004), University of Technology of Troyes, 10004 Troyes, France; mondher.rtimi@utt.fr (M.R.); nour.beydoun@utt.fr (N.B.); movsesyan@gmail.com (A.M.); sergei.kostcheev@utt.fr (S.K.); xavier.gassmann@utt.fr (X.G.)
2 Faculty of Sciences of Tunis, University of Tunis El Manar, Tunis 2092, Tunisia
3 Research and Technology Centre of Energy, Borj Cedria B.P N° 95, Hammam-Lif 2050, Tunisia; mohamed.lajnef@gmail.com (M.L.); radhouane.chtourou@crten.rnrt.tn (R.C.)
4 LCP-A2MC Laboratory, Jean Barriol Institute, University of Lorraine, 1 Bd Arago, 57070 Metz, France; suzanna.akil@univ-lorraine.fr
* Correspondence: safi.jradi@utt.fr

S1 Seed layers

Some previews work reported that the ZnO seed layers consisted of sequential layers deposited by repeating the dip coating or spin coating process for several cycles, over than 5 times, to synthesize then the nanowires in a chemical bath [1–3]. To study its morphological aspect, the ZnO seed layers were observed with a scanning electronic microscope (SEM). For this purpose, samples were prepared in sequential layers of one cycle (Figure S1-A), three cycles (Figure S1-B) and five cycles (Figure S1-C). The sample deposited on a sequence of 5 cycles has the highest ridge and then the height of ridges decreases by reducing the number of cycles (Figure S1-A-B and C). The Figure S1-D shows a zoom-in a ridge and it is seen in the figure S1-E that nanowires follow ridge orientation. However, we notice that for only one iteration (Figure S1-C) the surface of the layer becomes flat, homogeneous and the ridges disappears as seen in the zoom (Figure S1-F).

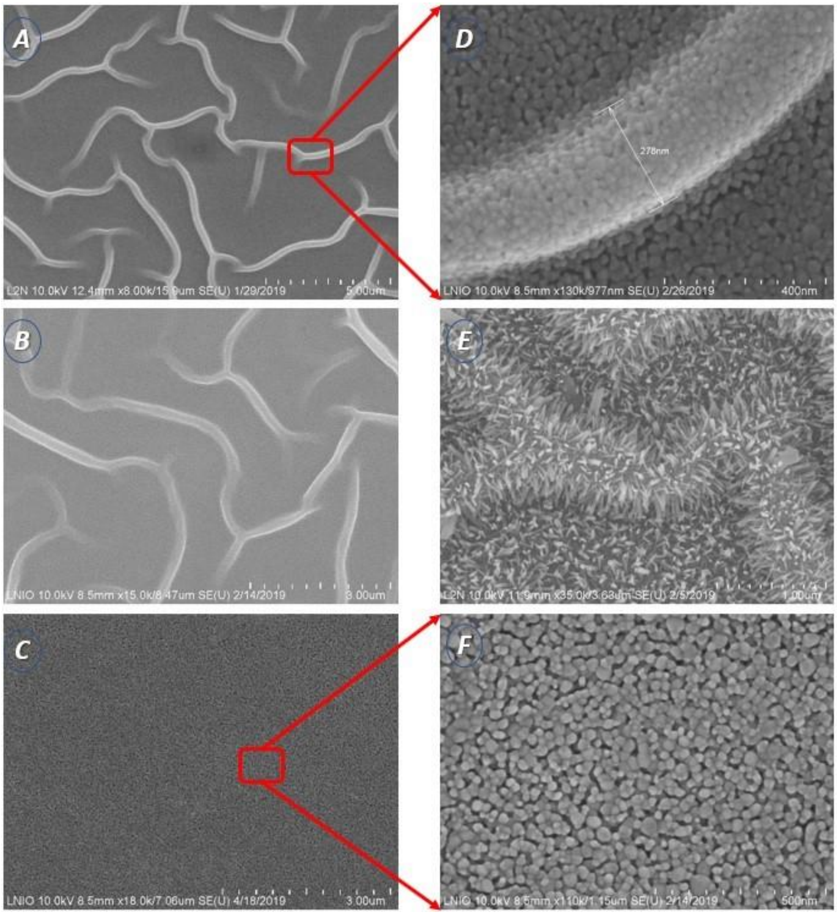

Figure S1. SEM images for the ZnO seed layers with different cycle of deposition procedure. (A) ZnO seed layers deposited in 5 cycles; (B) ZnO seed layers deposited in 3 cycles; (C) ZnO seed layer deposited in 1 cycle; (D) Zoom on a ridge; (E) ZnONWs grown on seed deposited in 5 cycles; (F) Zoom on a seed layer deposited in 1 cycle.

S2 Tauc plot

The energy band gap value could be calculated from the obtained UV-Visible absorption data using the Tauc formula given by [4, 5]:

$$(\alpha h\nu)^2 = (h\nu - E_g) \tag{1}$$

Where α is the absorption coefficient, h is the Planck constant, ν is the frequency and E_g is the band gap energy.

Figure S2 presents the result of $(\alpha h\nu)$ versus energy (eV) for ZnONWs and ZnONWs decorated by AlNSs. The band gap energy (E_g) value is the intersection point between the tangent to the linear part of the curve and the energy axis.

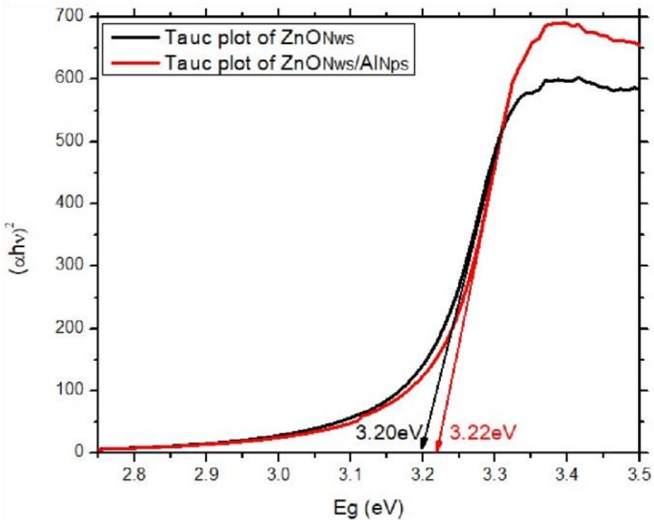

Figure S2. Tauc plot of ZnONws and ZnONws/AlNSs.

References

1. Bahramian, R.; Moshaii, A.; Eshghi, H. Effect of Seeding Modification of Substrate on the Growth and UV Detection Properties of ZnO Nanowires. *Materials Letters* **2016**, *179*, 222–225, doi:10.1016/j.matlet.2016.05.078.
2. Yoon, Y.-C.; Park, K.-S.; Kim, S.-D. Effects of Low Preheating Temperature for ZnO Seed Layer Deposited by Sol– Gel Spin Coating on the Structural Properties of Hydrothermal ZnO Nanorods. *Thin Solid Films* **2015**, *597*, 125–130, doi:10.1016/j.tsf.2015.11.040.
3. Ku, C.-H.; Wu, J.-J. Chemical Bath Deposition of ZnO Nanowire–Nanoparticle Composite Electrodes for Use in Dye-Sensitized Solar Cells. *Nanotechnology* **2007**, *18*, 505706, doi:10.1088/0957-4484/18/50/505706.
4. Saravanakkumar, D.; Sivaranjani, S.; Kaviyarasu, K.; Ayeshamariam, A.; Ravikumar, B.; Pandiarajan, S.; Veeralakshmi, C.; Jayachandran, M.; Maaza, M. Synthesis and Characterization of ZnO–CuO Nanocomposites Powder by Modified Perfume Spray Pyrolysis Method and Its Antimicrobial Investigation. *J. Semicond.* **2018**, *39*, 033001, doi:10.1088/1674-4926/39/3/033001.
5. Mittal, M.; Sharma, M.; Pandey, O.P. UV–Visible Light Induced Photocatalytic Studies of Cu Doped ZnO Nanoparticles Prepared by Co-Precipitation Method. *Solar Energy* **2014**, *110*, 386–397, doi:10.1016/j.solener.2014.09.026.
